# Future Self-Continuity and Grit in College Students: The Role of Future Time Perspective

**DOI:** 10.3390/bs15020144

**Published:** 2025-01-29

**Authors:** Dandan Wei, Yuting Ouyang, Xueyun Zeng

**Affiliations:** 1 School of Economics and Management, Beijing University of Posts and Telecommunications, Beijing 100876, China; dandanwei2024@163.com; 2 Faculty of Psychology, Beijing Normal University, Beijing 100875, China; yuting_ouyang@163.com

**Keywords:** future self-continuity, grit, future time perspective, college students

## Abstract

The present study examines the relationship between future self-continuity (FSC) and grit in college students, focusing on the mediating role of future time perspective (FTP) and its dimensions. A survey was conducted among 389 college students from universities in Beijing and Guizhou Province using relevant questionnaires. The results show the following: (1) FSC positively predicts grit (r = 0.25, *p* < 0.01); (2) a significant mediating role of FTP was found between FSC and grit; (3) and among the dimensions of FTP, behavioral commitment and future purpose consciousness significantly mediated the relationship between FSC and grit. This study demonstrates that FSC shapes college students’ grit through the mediation of future time perspective.

## 1. Introduction

In the context of high-quality development, grit has emerged as a crucial topic in the fields of youth mental health and educational psychology. College students, being in a critical stage of physical and psychological development, require grit—defined as an enduring passion and perseverance for long-term goals (Duckworth et al., 2007; J. Zhang et al., 2023). This quality significantly influences their future adaptability. But how can we cultivate and strengthen grit, which involves dedication and continuous effort toward achieving long-term aspirations? Grit, being a malleable quality (Duckworth et al., 2007; X. Li et al., 2023), can be developed. However, previous studies have predominantly focused on external factors such as autonomy-supportive environments (X. Li et al., 2023), parenting styles (T. Li et al., 2022), and social support (Ling et al., 2021), while insufficient attention has been given to the intrinsic psychological factors influencing grit development. While the primary focus of this study is the mediating role of future time perspective (FTP) in the relationship between future self-continuity (FSC) and grit, exploratory analyses were also conducted to examine potential differences in FSC and FTP across high- and low-grit groups. Previous studies have suggested that grit levels may influence future-oriented constructs (Zhao et al., 2022; Jiang & Lv, 2017), but these relationships remain underexplored. This analysis aims to provide a broader understanding of how these constructs interact in different subgroups.

## 2. Literature Review

### 2.1. Future Self-Continuity and Grit

From a psychological perspective, grit fundamentally represents an individual’s sustained enthusiasm and commitment to long-term goals (Duckworth et al., 2007). Theoretically, grit is conceptualized as a two-dimensional construct, consisting of perseverance of effort and consistency of interest. Therefore, it is closely linked to one’s future-oriented perspective, which requires exploration of future self-continuity (FSC). FSC refers to the degree to which an individual perceives a connection between their current self and their future self. It includes dimensions such as similarity, vividness, and positivity (Hershfield et al., 2009a; Hershfield, 2011). Individuals with higher FSC perceive their future self as a continuation of their present self, evaluate it positively, and demonstrate more future-oriented decisions and behaviors (Hershfield et al., 2009b). FSC helps to conserve these self-regulatory resources (“saving”) and acts as a resource supplement (“investment”) to support the development of grit. The core of self-regulation is resolving motivational conflicts (Baumeister & Vohs, 2007; Baumeister et al., 2007, 2016), as individuals inherently balance immediate needs and desires against the pursuit of long-term goals. Therefore, the key to developing grit lies in managing the conflict between short-term rewards and long-term goals. Individuals with higher FSC clearly understand how their current decisions and behaviors impact their future selves, prioritizing future benefits during decision-making (Liu et al., 2018). As a result, FSC reduces conflicts during the pursuit of long-term goals and alleviates the depletion of self-regulatory resources. Additionally, as a positive cognitive state, individuals with higher FSC possess greater self-regulatory resources, allowing them to resist temptations and exert self-control (Adelman et al., 2017; J. Zhang et al., 2023). Individuals with higher FSC also hold more positive evaluations of future outcomes (Hershfield et al., 2009a), demonstrating higher levels of delayed gratification and expectations for future results (Y. Yang & Zeng, 2022). Thus, they exhibit greater enthusiasm and determination when pursuing long-term goals.

Based on this, we propose the following:

**Hypothesis 1:**  *FSC positively predicts grit*.

### 2.2. The Mediating Role of Future Time Perspective

Given that both FSC and grit are constructs oriented toward the future, it is possible that a mediating mechanism related to future self-perception may exist between them. Future time perspective (FTP) refers to an individual’s cognitive, emotional, and behavioral orientations toward their future personal development and societal role (Zimbardo & Boyd, 1999; Zhao et al., 2022). FTP consists of five dimensions: behavioral commitment, which reflects the degree to which an individual commits to future goals through concrete actions; future purpose consciousness, characterized by a strong sense of purpose and direction regarding future aspirations; future efficacy, denoting an individual’s confidence in their ability to achieve future goals; far-reach goal orientation, involving the setting of long-term, ambitious objectives; and future image, which refers to the ability to vividly envision one’s future self and life circumstances (Song, 2004). Individuals with high FTP can clearly understand the link between current tasks and future goals, recognizing how present actions contribute to future outcomes (Simons et al., 2004). Theoretically, FSC is likely positively correlated with FTP, as individuals with high FSC, who tightly link their future self with their present self, are more aware of the impact of their current decisions on their future self. Previous studies have indirectly supported the relationship between FSC and FTP, indicating that college students with high FSC are more attentive to how current actions influence their future career paths (Adelman et al., 2017) and show higher expectations for future outcomes (Y. Yang & Zeng, 2022). They are also more inclined toward effective time management (F. Zhang et al., 2020), demonstrating higher levels of planning and time utilization, which in turn reflect elevated FTP.

Building on the self-regulation theory, this study explores the mechanism through which FTP mediates the relationship between FSC and grit. Future goals serve as cognitive representations of desired outcomes, and individuals with higher FTP are more adept at setting future goals, maintaining patience, and sustaining enthusiasm during the pursuit of these goals, thus continuously honing and reinforcing their grit (X. Yang et al., 2021; Song, 2004). FTP is closely linked to delayed gratification (Pang et al., 2014); individuals with higher FTP tend to invest time and effort into monitoring their progress toward goals and maintaining their pursuit of long-term aspirations. FTP enables individuals to anticipate the future consequences of their actions, fostering a positive outlook on the utility and outcomes of gritty behaviors and motivating the pursuit of long-term goals. Consequently, FTP acts as a self-regulatory mechanism, causing individuals to focus on future outcomes, guiding current behavior with future goals (Pang et al., 2014), and ultimately providing intrinsic motivation for developing grit. Previous studies have confirmed the positive association between FTP and grit: Jiang and Lv (2017) found that FTP positively predicts grit, while Zhao et al. (2022) demonstrated FTP’s mediating role in the relationship between growth mindsets and grit among college students.

Given these considerations, we propose the following:

**Hypothesis 2:**  *FTP mediates the relationship between FSC and grit*.

### 2.3. The Present Study

In summary, this study aims to investigate how FSC predicts grit among college students, with a focus on the mediating role of FTP. The findings are expected to reveal how FTP mediates the relationship between FSC and grit, offering a deeper understanding of the mechanisms underlying grit development in college students. This understanding will contribute to the existing body of research on self-regulation and motivation theories and provide practical guidance for fostering grit in educational settings.

## 3. Methods

### 3.1. Participants and Procedures

This study employed a survey-based approach. Upon obtaining informed consent from the participants, questionnaires were distributed using random sampling and targeting college students from universities in Beijing and Guizhou Province in 2022. A total of 485 questionnaires were collected, with 389 valid responses, resulting in an effective response rate of 80.20%. The participants’ ages ranged from 18 to 26 years, including 161 males and 228 females. The sample included 215 students from science and engineering majors, 169 from liberal arts majors, and 5 from arts and sports majors. Furthermore, 127 participants were only children, while 262 were not; 187 students came from urban areas, and 202 came from rural areas.

### 3.2. Measures

#### 3.2.1. Future Self-Continuity Questionnaire (FSCQ)

The FSCQ, developed by Sokol and Serper (2020), was used to measure future self-continuity. This questionnaire comprises three dimensions: positivity, vividness, and similarity, and includes a total of 10 items. Participants rated each item using a 7-point Likert scale (1 = “Completely disagree”; 7 = “Completely agree”). In this study, the overall score was used in the primary analyses, while subdimension scores were analyzed in exploratory analyses to understand their unique contributions. The internal consistency coefficient (Cronbach’s alpha) for the overall FSCQ was 0.85, indicating good reliability. The Cronbach’s alphas for the subdimensions were 0.81 (positivity), 0.78 (vividness), and 0.83 (similarity). A higher overall score indicates a stronger perceived connection between one’s present and future self.

#### 3.2.2. Future Time Perspective Scale (FTPS)

The FTPS, developed by Song (2004), was administered to assess the future time perspective of college students. The scale consists of five dimensions: behavioral commitment, future efficacy, far-reach goal orientation, future purpose consciousness, and future image, encompassing a total of 20 items. A 5-point Likert scale was used for scoring. In this study, both the overall score of the FTPS and the scores of its five subdimensions were analyzed. The internal consistency coefficient (Cronbach’s alpha) for the overall FTPS was 0.88, indicating excellent reliability. For the subdimensions, Cronbach’s alpha values were as follows: behavioral commitment (0.78), future efficacy (0.83), far-reach goal orientation (0.81), future purpose consciousness (0.76), and future image (0.75), all of which demonstrate acceptable to good reliability. Higher scores on the FTPS or its subdimensions indicate a stronger future-oriented perspective.

#### 3.2.3. Grit Scale

The Grit Scale, formulated by Duckworth et al. (2007), was utilized to evaluate participants’ grit. The scale includes two dimensions: “perseverance of effort” and “consistency of interest”, with a total of 12 items. It employs a 5-point Likert scale for scoring. The perseverance of effort dimension consists of 6 positively scored items, while the consistency of interest dimension includes 6 negatively scored items, which were reverse-coded. In this study, the overall score was used as the primary measure of grit, while subdimension scores were included in exploratory analyses. The internal consistency coefficient (Cronbach’s alpha) for the overall Grit Scale was 0.73, indicating satisfactory reliability. Cronbach’s alphas for perseverance of effort and consistency of interest were 0.77 and 0.70, respectively. A higher score indicates a higher level of grit, reflecting greater passion and perseverance for long-term goals.

### 3.3. Statistical Analysis

Data analysis was conducted using SPSS 26.0. The following statistical methods were applied: Common Method Bias Test: Harman’s single-factor test was used to check for common method bias. Descriptive Statistics and Correlation Analysis: These analyses were conducted to explore the relationships between FSC, FTP, and grit among college students. Mediation Analysis: Hayes’ PROCESS 4.0 macro for SPSS (Model 4) was employed to examine the mediating role of FTP. The bias-corrected percentile Bootstrap method was used to test the significance of the mediation effect.

## 4. Results

### 4.1. Measurement Reliability and Validity

In this study, the internal consistency and validity of the questionnaires were examined using SPSS 26.0. Cronbach’s alpha coefficients for all dimensions of the scales ranged from 0.70 to 0.88, indicating higher internal consistency. The Kaiser–Meyer–Olkin (KMO) measure of sampling adequacy for the questionnaires was above 0.77, demonstrating good construct validity. The cumulative variance percentages for FSC, FTP, and grit were 73.56%, 83.65%, and 71.79%, respectively, highlighting the strong explanatory power of the scales.

To test for common method bias, Harman’s single-factor test was applied (Zhou & Long, 2004). The results indicated that the variance explained by the first factor was 24.78%, which is below the 40% threshold, and the number of factors with eigenvalues greater than 1 was 10, explaining 64.52% of the total variance. Therefore, there is no significant issue with common method bias in this study.

### 4.2. FSC and FTP Across Grit Groups

As part of an exploratory analysis, this study examined the differences in FSC and FTP across high- and low-grit groups to better understand how these constructs interact in individuals with varying levels of grit. Participants were divided into two groups based on their grit scores: those at or below the 27th percentile were classified as the low-grit group, while those at or above the 73rd percentile constituted the high-grit group. Independent sample t-tests were conducted to compare FSC and FTP between these groups (Table 1). The results revealed significant differences, with the high-grit group demonstrating higher levels of both FSC and FTP compared to the low-grit group (*p* < 0.05).

### 4.3. Pearson Correlation Analysis

Correlation analyses were conducted to examine the relationships between the overall scores of FSC, FTP, and grit, as well as their respective subdimensions (see Table 2).

This approach aimed to provide a detailed understanding of how each dimension contributes to the overall relationships. Mediation analyses were also performed for both the overall FTP and its five subdimensions to explore their unique roles in mediating the relationship between FSC and grit. The mean grit score among college students was 3.13 (SD = 0.45), indicating a moderately high level of grit. FSC had a mean score of 4.41 (SD = 0.96), suggesting a high level among the participants. The mean score for FTP was 3.55 (SD = 0.49), demonstrating a moderately high level. About the correlation relationship, FSC was positively correlated with grit (r = 0.245, *p* < 0.01), as well as with FTP (r = 0.404, *p* < 0.01). FTP was also positively correlated with grit (r = 0.528, *p* < 0.01).

These findings suggest that higher levels of FSC and FTP are associated with greater grit among college students, highlighting the potential importance of temporal perspectives in fostering perseverance and passion for long-term goals.

### 4.4. FSC and Grit: Direct Effect and Mediation Analysis

Hayes’ PROCESS 4.0 macro for SPSS was used to test the mediating effect of FTP between FSC and grit (Hayes, 2013). Gender, age, and academic year were controlled in the analysis, and the Bootstrap 95% confidence interval was calculated. The regression analysis results (see Table 3) showed that FSC positively predicted both FTP (β = 0.40, t = 8.59, *p* < 0.001) and grit (β = 0.23, t = 4.70, *p* < 0.001), supporting Hypothesis 1.

When the mediating variable (FTP) was controlled for, the direct effect of FSC on grit was not significant; meanwhile, FTP positively predicted grit (β = 0.51, t = 10.85, *p* < 0.001), validating Hypothesis 2.

The mediation model further confirmed that the indirect effect of FSC on grit through FTP was significant (indirect effect value = 0.21, 95% CI = [0.14, 0.27]), while a direct effect did not exist (effect value = 0.02, 95% CI = [−0.07, 0.12], *p* > 0.05; see Table 4). This indicates that the effect of FSC on grit is fully mediated by FTP, suggesting that individuals with a stronger sense of FSC are more likely to develop grit indirectly by fostering a stronger FTP. The total effect of FSC on grit was significant (effect value = 0.23, 95% CI = [0.13, 0.32], *p* < 0.001), further emphasizing the importance of FTP as a crucial intermediary. These results provide strong support for the hypothesis and highlight the key role of FTP in linking FSC to grit. 

### 4.5. The Mediating Role of FTP in Shaping Grit

**Figure 1:**
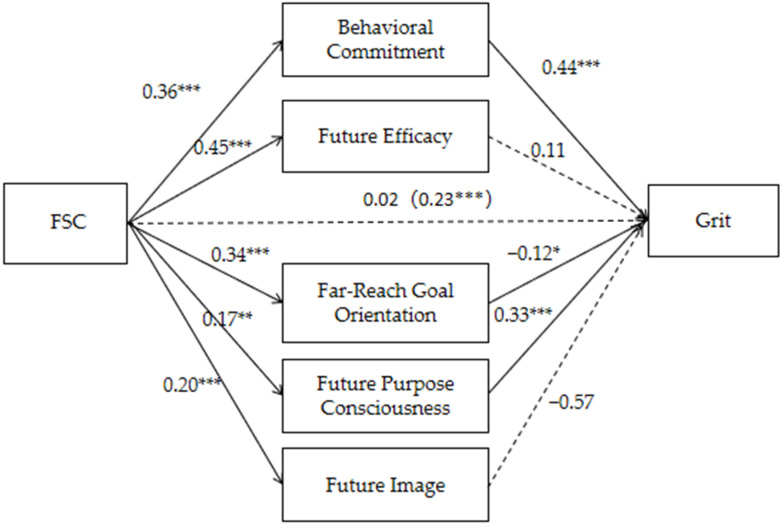
Mediation model of FTP between FSC and grit. * *p* < 0.05, ** *p* < 0.01, *** *p* < 0.001.

To further explore the mediating path of FTP, a mediation model was constructed with FSC as the independent variable, grit as the dependent variable, and the five sub-dimensions of FTP—behavioral commitment, future efficacy, far-reach goal orientation, future purpose consciousness, and future image—as mediating variables. As shown in Figure 1 and Table 5, FSC has a significant total effect on grit (β = 0.23, *p* < 0.001).

FSC positively predicts behavioral commitment (β = 0.36, *p* < 0.001), and behavioral commitment positively predicts grit (β = 0.44, *p* < 0.001). FSC positively predicts future purpose consciousness (β = 0.17, *p* < 0.001), and future purpose consciousness positively predicts grit (β = 0.33, *p* < 0.001). But the mediating effects of far-reach goal orientation, future image, and future efficacy do not exist.

The results (see Table 6) show that behavioral commitment plays a significant full mediating role in the relationship between FSC and grit, and future purpose consciousness also serves as a full mediator in this relationship. However, the mediating effects of future efficacy, far-reach goal orientation, and future image are not significant. Specifically, as mediating variables, behavioral commitment and future purpose consciousness significantly enhance the positive impact of FSC on grit, with standardized indirect effect values of 0.16 and 0.06, respectively. Their 95% confidence intervals do not contain zero, confirming their significant positive mediating effect.

In contrast, the mediating effects of future efficacy, far-reach goal orientation, and future image were not significant. For these dimensions, the 95% confidence intervals included zero, suggesting no valid indirect effects.

## 5. Discussion

### 5.1. Summary of the Findings

This study, based on self-regulation theory, reveals the underlying mechanism through which FSC influences grit. Self-regulation theory posits that individuals adjust their behavior based on their cognition and self-expectations. Individuals with higher FSC view their present self and future self as a coherent whole. This cognitive perspective provides them with stronger motivation in the self-regulation process to make decisions that benefit their future (Hershfield et al., 2009b; Simons et al., 2004; Pang et al., 2014; Wang et al., 2020).

This study also found that FTP fully mediates the relationship between FSC and grit in college students. This finding supports Baumeister and Oettingen’s model (2016). Baumeister’s two-stage model of future thinking suggests that when individuals think about the future, they go through two stages: the first stage involves anticipating future outcomes, while the second stage focuses on planning and acting to achieve those outcomes. FSC enables college students to form positive expectations about the future, prompting them to consider how their current actions and decisions impact the future, thereby fostering grit.

In the discussion, we emphasize that, while all five dimensions of FTP were explored in the current study, only behavioral commitment and future purpose consciousness were found to significantly mediate the relationship between FSC and grit. The other dimensions, such as future efficacy, far-reach goal orientation, and future image, did not exhibit mediating effects. Behavioral commitment reflects the degree of commitment individuals have toward future actions. The study found that individuals with higher FSC exhibit higher levels of behavioral commitment, which is closely related to the consistency and stability of grit. When individuals make a clear commitment to achieving long-term goals, they are more likely to mobilize the necessary self-regulatory resources to overcome obstacles encountered in the pursuit of those goals. Future purpose consciousness reveals the degree to which individuals recognize the meaning and importance of their goals. Individuals with higher FSC often have a clearer understanding of their goals and the meaning behind pursuing them, which fuels their intrinsic motivation and encourages them to maintain enthusiasm and persistence when facing challenges (Wang et al., 2020). Future purpose consciousness is also closely associated with goal-directed behavior, helping individuals stay focused on long-term goals even in the face of short-term temptations.

### 5.2. Theoretical Significance and Practical Implications

#### 5.2.1. Theoretical Significance

This study explores the impact of FSC on grit in college students and the potential mediating mechanism of FTP. Firstly, it deepens the understanding of the factors influencing grit. This study reveals that FSC influences grit through the mediating variable of FTP, emphasizing the key role of future time perspective in the development of grit. Secondly, this study expands the research on FSC. Previous studies mainly focused on explaining how FSC affects positive behaviors, such as improving self-control (J. Zhang et al., 2023), supporting intertemporal risk decisions (Bartels & Urminsky, 2011), and increasing academic engagement (Rutchick et al., 2018). This study shifts the perspective from behavior to character shaping. FSC plays a crucial role in reducing the depletion of self-regulatory resources. This protection of self-resources makes individuals have more grit in pursuing long-term goals. Thirdly, this study elaborates on how FTP shapes grit through self-regulation mechanisms such as goal setting, monitoring, and execution. This provides empirical support for self-regulation theory in explaining the pursuit of long-term goals and character, thereby enhancing the application of self-regulation theory in this domain.

#### 5.2.2. Practical Implications

From the educational practice perspective, this research offers important insights. Educators can use course design and teaching activities to help students establish the combination between their present self and future self. For example, activities such as writing letters to their future selves, setting long-term goals, and planning career paths can help students gain a deeper understanding of the long-term impact of their current behaviors. Educational practices should encourage students to make clear commitments to their academic objectives and activities. By setting clear, measurable goals and tracking their progress, students can strengthen their commitment to these goals, which will, in turn, help them develop greater grit when facing challenges. This consciousness not only contributes to students’ academic improvement but also fosters a more resilient character in their personal growth.

### 5.3. Limitations and Future Directions

This study is cross-sectional, and future research could employ experimental designs or longitudinal tracking to further explore the impact of FSC on grit. The data used in this study were all self-reported, which may introduce social desirability bias and common method variance. Future research could use multi-source reports to assess grit to reduce measurement bias. Furthermore, future research could focus on the role of social support, including assistance from university psychological centers, courses, and mentors, in strengthening FSC and FTP.

## 6. Conclusions

In summary, this study reveals the relationship between college students’ FSC, FTP, and grit. FSC positively predicts grit, with FTP fully mediating the relationship between FSC and grit. Specifically, behavioral commitment and future purpose consciousness serve as significant full mediators, while the mediating effects of future efficacy, far-reach goal orientation, and future image do not exist. The findings explain the process of grit formation, expanding the positive significance of FSC. It also provides insights into the cultivation and shaping of college students’ grit in educational practice.

## Figures and Tables

**Table 1:** FSC and FTP across grit groups (N = 389).

Variable	Low Group (n = 128)	High Group (n = 128)	t	Cohen’s d	Degrees of Freedom
	M ± SD	M ± SD			
1 Positivity	4.10 ± 1.08	4.10 ± 1.08	−2.374 **	0.30	251
2 Vividness	3.67 ± 1.31	4.35 ± 1.29	−4.167 ***	0.53	251
3 Similarity	4.57 ± 1.35	5.35 ± 1.32	−4.652 ***	0.58	251
4 Future Self-Continuity	4.11 ± 0.90	4.69 ± 0.98	−4.905 ***	0.63	251
5 Behavioral Commitment	3.34 ± 0.61	4.01 ± 0.51	−9.441 ***	1.19	251
6 Future Efficacy	3.52 ± 0.79	4.14 ± 0.60	−7.053 ***	0.92	251
7 Far-Reach Goal Orientation	3.42 ± 0.68	3.80 ± 0.64	−4.662 ***	0.60	251
8 Future Purpose Consciousness	2.69 ± 0.65	3.54 ± 0.76	−9.59 ***	1.37	251
9 Future Image	3.61 ± 0.60	3.92 ± 0.54	−4.274 ***	0.54	251
10 Future Time Perspective	3.31 ± 0.48	3.86 ± 0.46	−9.519 ***	1.22	251

Note: ** *p* < 0.01, *** *p* < 0.001.

**Table 2:** Correlation analysis (N = 389).

Variables	M (SD)	FSC1	FSC2	FSC3	FSC	FTP1	PTP2	FTP3	FTP4	FTP5	FTP	Grit
FSC1	4.28 (1.16)	1										
FSC2	4.09 (1.30)	0.444 **	1									
FSC3	4.91 (1.32)	0.233 **	0.452 **	1								
FSC	4.41 (0.96)	0.765 **	0.813 **	0.712 **	1							
FTP1	3.65 (0.61)	0.201 **	0.315 **	0.344 **	0.369 **	1						
FTP2	3.81 (0.72)	0.212 **	0.339 **	0.493 **	0.446 **	0.671 **	1					
FTP3	3.62 (0.64)	0.157 **	0.335 **	0.309 **	0.342 **	0.663 **	0.606 **	1				
FTP4	3.00 (0.81)	0.01 **	0.136 **	0.283 **	0.178 **	0.318 **	0.337 **	0.200 **	1			
FTP5	3.72 (0.57)	0.067 **	0.147 **	0.272 **	0.205 **	0.439 **	0.520 **	0.441 **	0.359 **	1		
FTP	3.55 (0.49)	0.165 **	0.338 **	0.448 **	0.404 **	0.812 **	0.810 **	0.786 **	0.626 **	0.713 **	1	
Grit	3.13(0.45)	0.109 *	0.203 **	0.264 **	0.245 **	0.528 **	0.417 **	0.287 **	0.472 **	0.262 **	0.528 **	1

Note: * *p* < 0.05, ** *p* < 0.01. Positivity (FSC1), vividly (FSC2), similarity (FSC3), future self-continuity (FSC), behavioral commitment (FTP1), future efficacy (FTP2), far-reach goal orientation (FTP3), future purpose consciousness (FTP4), future image (FTP5), future time perspective (FTP), and grit.

**Table 3:** Direct effect and mediation analysis (N = 389).

Regression Equation		Overall Fit Index			Coefficient Significance	
Dependent Variables	Predictor	R	R^2^	F	β	t
1 Grit		0.30	0.09	9.56 ***		
	Gender				−0.08	−1.71
	Age				0.15	2.54 **
	Academic Year				−0.00	−0.08
	FSC				0.23	4.70 ***
2 FTP		0.42	0.18	20.76 ***		
	Gender				0.07	1.41
	Age				0.11	1.90
	Academic Year				−0.01	−0.19
	FSC				0.40	8.59 ***
3 Grit		0.55	0.30	33.51 ***		
	Gender				−0.12	−2.73 **
	Age				0.10	1.85
	Academic Year				0.00	0.02
	FTP				0.51	10.85 ***
	FSC				0.02	0.52

Note: All variables in the model were standardized., ** *p* < 0.01, *** *p* < 0.001.

**Table 4:** The effects of FSC on grit through FTP (N = 389).

Effect Type	Path	Effect Value	Bootstrapped Standard Error	95% Confidence Interval
Total Effect		0.23	0.05	[0.13, 0.32]
Direct Effect	FSC → Grit	0.02	0.05	[−0.07, 0.12]
Indirect Effect	FSC → FTP → Grit	0.21	0.03	[0.14, 0.27]

**Table 5:** Analysis of the mediating role of FTP between FSC and grit (N = 389).

Regression Equation		Overall Fit Index			Coefficient Significance	
Dependent Variable	Predictor	R	R^2^	F	β	t
1 Grit		0.30	0.09	9.56 ***		
	FSC				0.23	4.66 ***
2 Behavioral Commitment		0.39	0.16	17.77 ***		
	FSC				0.36	7.63 ***
3 Future Efficacy		0.46	0.21	26.09 ***		
	FSC				0.45	9.86 ***
4 Far-Reach Goal Orientation		0.35	0.12	13.25 ***		
	FSC				0.34	7.13 ***
5 Future Purpose Consciousness		0.24	0.06	6.02 ***		
	FSC				0.17	3.37 **
6 Future Image		0.22	0.05	4.73 ***		
	FSC				0.20	4.07 ***
7 Grit		0.64	0.41	29.09 ***		
	Behavioral Commitment				0.44	7.31 ***
	Future Efficacy				0.11	1.75
	Far-Reach Goal Orientation				−0.12	−2.11 *
	Future Purpose Consciousness				0.33	7.53 ***
	Future Image				−0.57	−1.17
	FSC				0.02	0.38

Note: * *p* < 0.05, ** *p* < 0.01, *** *p* < 0.001.

**Table 6:** Mediation test of the five dimensions of FTP between FSC and grit (N = 389).

Effect	Path		Standardized Effect Size	Bootstrapped Standard Error	95% Confidence Interval	
					Lower Limit	Upper Limit
Total Effect			0.23	0.05	0.13	0.32
Direct Effect	FSC→Grit		0.02	0.05	−0.07	0.11
Indirect Effect	FSC→Behavioral Commitment→Grit		0.16	0.04	0.09	0.23
	FSC→Future Efficacy→Grit		0.05	0.04	−0.03	0.12
	FSC→Far-Reach Goal Orientation→Grit		−0.04	0.03	−0.09	0.02
	FSC→Future Purpose Consciousness→Grit		0.06	0.02	0.02	0.10
	FSC→Future Image→Grit		−0.01	0.01	−0.04	0.01
Total Indirect Effect			0.21	0.04	0.13	0.29

## Data Availability

The data are not publicly available due to privacy.
